# Listeria monocytogenes Sublethal Injury and Viable-but-Nonculturable State Induced by Acidic Conditions and Disinfectants

**DOI:** 10.1128/Spectrum.01377-21

**Published:** 2021-12-15

**Authors:** Marianna Arvaniti, Panagiotis Tsakanikas, Vasiliki Papadopoulou, Artemis Giannakopoulou, Panagiotis Skandamis

**Affiliations:** a Laboratory of Food Quality Control and Hygiene, Department of Food Science and Human Nutrition, Agricultural University of Athens, Athens, Greece; b Laboratory of Microbiology and Biotechnology of Foods, Department of Food Science and Human Nutrition, Agricultural University of Athens, Athens, Greece; University of Torino

**Keywords:** *Listeria monocytogenes*, VBNC state, acid stress, disinfectants, peracetic acid, sublethal injury

## Abstract

The dormancy continuum hypothesis states that in response to stress, cells enter different stages of dormancy ranging from unstressed living cells to cell death, in order to ensure their long-term survival under adverse conditions. Exposure of Listeria monocytogenes cells to sublethal stressors related to food processing may induce sublethal injury and the viable-but-nonculturable (VBNC) state. In this study, exposure to acetic acid (AA), hydrochloric acid (HCl), and two disinfectants, peracetic acid (PAA) and sodium hypochlorite (SH), at 20°C and 4°C was used to evaluate the potential induction of L. monocytogenes strain Scott A into different stages of dormancy. To differentiate the noninjured subpopulation from the total population, tryptic soy agar with 0.6% yeast extract (TSAYE), supplemented or not with 5% NaCl, was used. Sublethally injured and VBNC cells were detected by comparing plate counts obtained with fluorescence microscopy and by using combinations of carboxyfluorescein and propidium iodide (viable/dead cells). Induction of sublethal injury was more intense after PAA treatment. Two subpopulations were detected, with phenotypes of untreated cells and small colony variants (SCVs). SCVs appeared as smaller colonies of various sizes and were first observed after 5 min of exposure to 5 ppm PAA at 20°C. Increasing the stress intensity from 5 to 40 ppm PAA led to earlier detection of SCVs. L. monocytogenes remained culturable after exposure to 20 and 30 ppm PAA for 3 h. At 40 ppm, after 3 h of exposure, the whole population was considered nonculturable, while cells remained metabolically active. These results corroborate the induction of the VBNC state.

**IMPORTANCE** Sublethally injured and VBNC cells may evade detection, resulting in underestimation of a food product’s microbial load. Under favorable conditions, cells may regain their growth capacity and acquire new resistant characteristics, posing a major threat for public health. Induction of the VBNC state is crucial for foodborne pathogens, such as L. monocytogenes, the detection of which relies almost exclusively on the use of culture recovery techniques. In the present study, we confirmed that sublethal injury is an initial stage of dormancy in L. monocytogenes that is followed by the VBNC state. Our results showed that PAA induced SCVs (a phenomenon potentially triggered by external factors) and the VBNC state in L. monocytogenes, indicating that tests of lethality based only on culturability may provide false-positive results regarding the effectiveness of an inactivation treatment.

## INTRODUCTION

Listeria monocytogenes is a Gram-positive foodborne bacterial pathogen that is often found in nature and in food-processing environments and has a high mortality rate, reaching 20 to 30% (1). It has been characterized as an opportunistic pathogen with various degrees of virulence and pathogenicity at the strain level (2, 3). The microorganism has an increased survival capacity against adverse environmental conditions (4) and the ability to adapt to a wide range of conditions, such as refrigeration, low pH, and high salinity, as well as the host environment (5).

Bacteria are exposed to different stressors, covering the production-to-digestion continuum (6). In suboptimal environments, the transition of bacterial cells between different physiological states, ranging from unstressed living cells to deep dormancy and cell death (7, 8), reveals the need to distinguish viability from culturability (9). Sublethal injury is the result of one or more sublethal treatments, including chemical or physical processes, that damage but do not kill a microorganism (10, 11). Depending on the intensity and duration of exposure to stress, the injury may be temporary, causing sublethal damage, or permanent, leading to death (7). One additional consequence of this state is the loss of culturability. Due to cell damage, sublethally injured cells become sensitive to selective agents (6). The viable-but-nonculturable (VBNC) state is a survival strategy (12) that provides long-term bacterial survival under unfavorable conditions (13, 14) like suboptimum growth temperatures, nutrient starvation, increased salinity (15), low pH (16), and osmotic stress (13). Also, exposure to food preservatives (13) and chlorination (17, 18) may induce the VBNC state. The main characteristic of VBNC cells is that they fail to form colonies on nutrient-rich, nonselective bacteriological media but retain their metabolic activity and may become culturable upon resuscitation (12). Sublethally injured cells that remain metabolically active but cannot be resuscitated in culture media may enter the VBNC state (11).

In food-processing facilities, L. monocytogenes may become persistent (19), surviving the cleaning and disinfection procedures and frequently reoccurring in different niches and final products (20), leading to low conformity with regulatory standards and a potential public health threat (21). A subpopulation of the original population may have the ability to survive after exposure to food-related lethal stresses like heat, high hydrostatic pressure (HHP), and antimicrobials. If this fraction of cells has the ability to generate a new population equally as sensitive to stressors as the ancestral population, they are called persisters (20). According to the microbial “dormancy continuum hypothesis,” persisters are in the initial stages of dormancy, with quick resuscitation potential upon the removal of stress, while VBNC cells are in a deeper stage of dormancy (22). Persistence is a state of dormancy, stochastically formed, where a proportion of cells transiently becomes multidrug tolerant (23). It has been associated with exposure to different kinds of stress, including starvation, carbon source depletion, oxidative stress, pH stress, and DNA damage (24–28). Alterations in the colony morphotype may reveal altered cellular phenotypes, which can contribute to persistence in a natural setting (29).

Induction of the VBNC state is crucial for foodborne pathogens, whose detection relies almost exclusively on the use of culture-based techniques for recovery (30). This reveals a need for alternate, non-culture-based methods able to evaluate cellular viability. Taking into account that the VBNC state represents an important reservoir of human pathogens in natural environments (31), it is clear that a combination of different methods that indicate culturability and metabolic activity must be used in parallel, in order to evaluate product safety and prevent potential foodborne diseases. Several studies have reported induction of *Listeria* spp. into the VBNC state (16, 32–34). However, relatively little information exists regarding either the transition of L. monocytogenes among sublethal injury, persistence, and the VBNC state or comparative assessment of population-level phenotypic behavior and the different physiological states of single cells. In the present study, we evaluated L. monocytogenes survival and potential for induction into different stages of dormancy after exposure to acid stress (acetic acid [AA] and hydrochloric acid [HCl]) and to two different disinfectants (peracetic acid [PAA] and sodium hypochlorite [SH]) at 20°C (ambient conditions in food processing environments) and 4°C (refrigeration temperature). We focused on stress conditions that are related to food-processing environments, such as acid stress, the combined biological effect of low pH and weak (organic) acids, and common disinfectants used in food equipment sanitation, such as peracetic acid (35). Acidification of foods is a means of food preservation that remains a principle hurdle to control the outgrowth of pathogens (36). In order to distinguish the VBNC fraction, we used plate counting and fluorescence microscopy in parallel. The reasoning for using those approaches together is that the first allows us to assess the phenotypic response of the population to the stress applied, while the latter enables us to enumerate the different states induced at the single-cell level. The objectives of the present study were (i) to examine sublethal injury at the single-cell level and the population level, (ii) to evaluate alterations in colony morphotype that indicate persistence, and (iii) to outline the proportions of culturable, injured, VBNC, and dead cells using fluorescence microscopy and carboxyfluorescein diacetate (CFDA)/propidium iodide (PI) double staining.

## RESULTS

### Sublethal injury of L. monocytogenes after exposure to AA and HCl.

**FIG 1:**
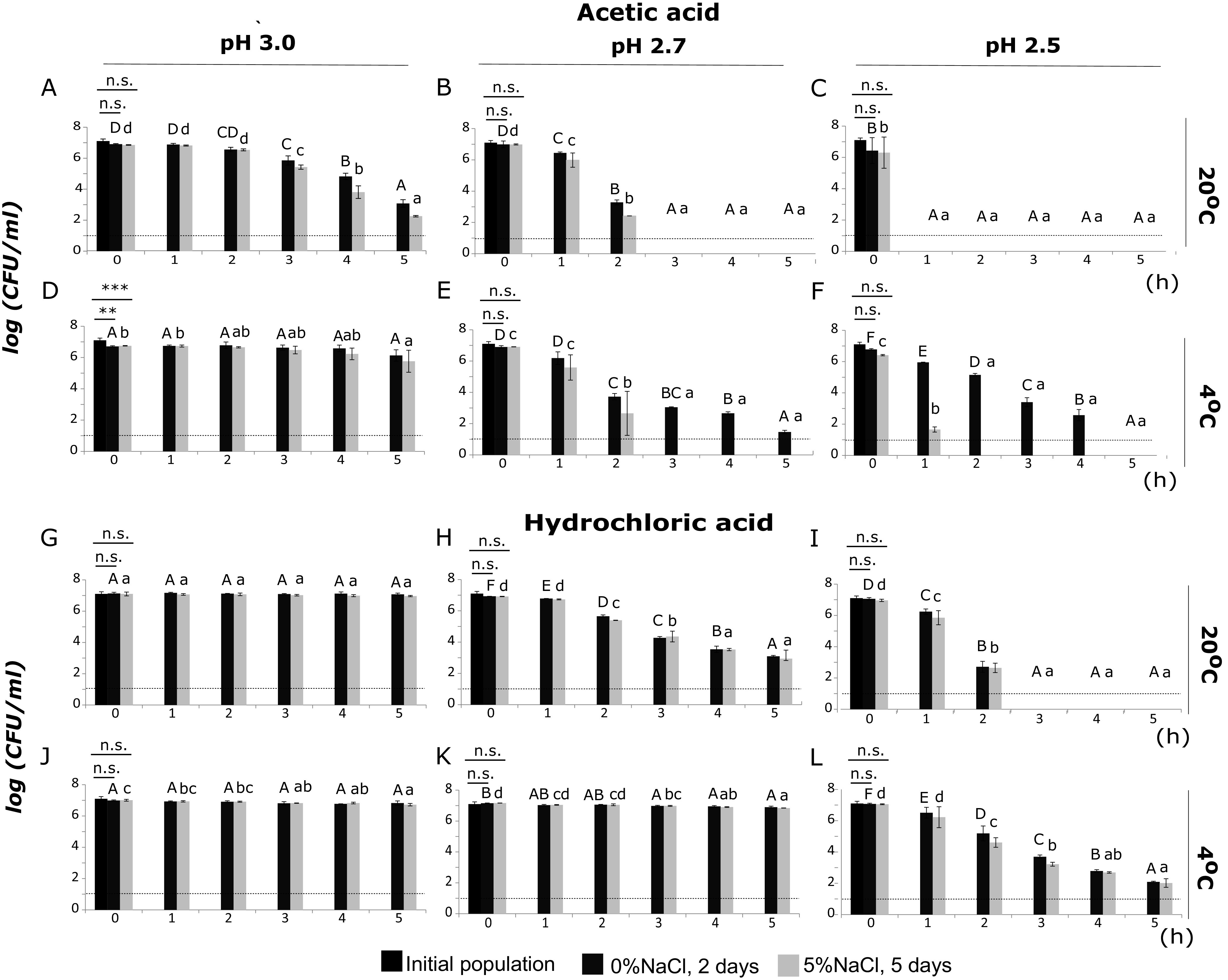
Populations (log CFU/ml) of L. monocytogenes, strain Scott A, after incubation for 0, 1, 2, 3, 4, or 5 h under the following conditions: (A) AA, pH 3.0, at 20°C, (B) AA, pH 2.7, at 20°C, (C) AA, pH 2.5, at 20°C, (D) AA, pH 3.0, at 4°C, (E) AA, pH 2.7, at 4°C, (F) AA, pH 2.5, at 4°C, (G) HCl, pH 3.0, at 20°C, (H) HCl, pH 2.7, at 20°C, (I) HCl, pH 2.5, at 20°C, (J) HCl, pH 3.0, at 4°C, (K) HCl, pH 2.7, at 4°C, and (L) HCl, pH 2.5, at 4°C. Total culturable (dark gray bars) and noninjured (light gray bars) population levels were measured after incubation for 2 days and 5 days at 37°C on TSAYE supplemented with 0% and 5% NaCl, respectively. Bars represent mean values ± standard deviations (SD) from 3 independent biological replicates. Statistically significant differences (*P* < 0.05) are indicated with different uppercase (TSAYE) and lowercase (TSAYE+5%NaCl) letters, respectively. The dotted line represents the limit of detection (LOD). **, *P* < 0.05; ***, *P* < 0.01; n.s., not significant.

For acetic acid, statistical analysis of the differences between the mean total culturable populations (enumerated on tryptic soy agar with 0.6% yeast extract [TSAYE]) at the three levels of pH (3.0, 2.7, and 2.5) at the same time of exposure and temperature (20°C or 4°C) revealed different inactivation patterns over time (Table S1 in the supplemental material). Statistically significant differences in total culturable populations were detected after incubation for 2 h at 20°C and 4°C (*P* < 0.05). Incubation for 5 h at 20°C in Ringer’s solution at pH 3.0, adjusted with AA, resulted in a 3.8-log reduction of the total culturable population (Fig. 1A). The population of noninjured cells (enumerated on TSAYE supplemented with 5% NaCl [TSAYE+5%NaCl]) at the same time decreased by up to 4.6 log units (Fig. 1A). The highest levels of sublethal injury were observed within 3 to 5 h of incubation. The population levels in TSAYE and TSAYE+5%NaCl after exposure to pH 3.0 for 3, 4, and 5 h at 20°C were significantly higher than those after exposure to pH 2.7 and 2.5, since the latter were below the limit of detection (LOD) of 1 log CFU/ml. After 5 h of exposure to pH 3.0 (AA) at 4°C, the total culturable population remained at approximately 6 log CFU/ml (Fig. 1D), significantly higher than the total culturable populations detected at pH 2.7 and 2.5 (Table S1). After 2 h at 20°C in Ringer’s solution with pH 2.7, adjusted with AA, the total culturable population decreased from 6.9 to 3.2 log CFU/ml and the population on TSAYE+5%NaCl was reduced to 2.4 log CFU/ml (Fig. 1B). From 3 to 5 h of exposure under the same conditions, the total culturable population and the noninjured population were below the detection limit (Fig. 1B). In contrast, the total culturable population was detectable after incubation for 5 h at 4°C at pH 2.7, adjusted with AA (Fig. 1E). After 2 h of exposure, the number of injured cells was increased. Finally, when cells were exposed to pH 2.5, adjusted with AA, at 20°C, the populations of L. monocytogenes (total and noninjured) fell below the enumeration limit from the first sampling point (Fig. 1C), while at 4°C, there was a clear trend of injury until 4 h of incubation (Fig. 1F).

Sublethal injury and inactivation were evaluated after exposure to the same pH values (3.0, 2.7, and 2.5), adjusted with HCl, at 20°C and 4°C. More specifically, when 10^7^ CFU/ml cells were exposed to pH 3.0 adjusted with HCl for 5 h at 20°C and 4°C, the total culturable populations remained at the same level throughout the exposure (Fig. 1G and J). Following 2, 3, 4, and 5 h of exposure at 20°C, the total culturable populations, as well as the populations of noninjured cells, showed significant differences between the 3 levels of pH (Table S2). Exposure to pH 2.7 (adjusted with HCl) caused decreases of up to 3.8 log units in the total culturable population and 3.9 log units in the noninjured population after 5 h of incubation at 20°C (Fig. 1H). When the challenge pH was reduced further, to 2.5, the total culturable population, as well as the population enumerated on TSAYE+5%NaCl, showed a significant decrease of up to 4.3 log after 2 h of stress exposure at 20°C (*P* < 0.05) (Fig. 1I). Furthermore, it is significant to report that at time zero (within 10 s) of exposure at 4°C, the L. monocytogenes populations (total culturable and noninjured) were different at pH 3.0 and 2.5 (Table S2). Incubation for 5 h at 4°C at pH 2.5 adjusted with HCl resulted in 4.9- and 5.0-log reductions of the total culturable population and the noninjured population, respectively (Fig. 1L).

### Sublethal injury of L. monocytogenes after exposure to PAA and SH.

**FIG 2:**
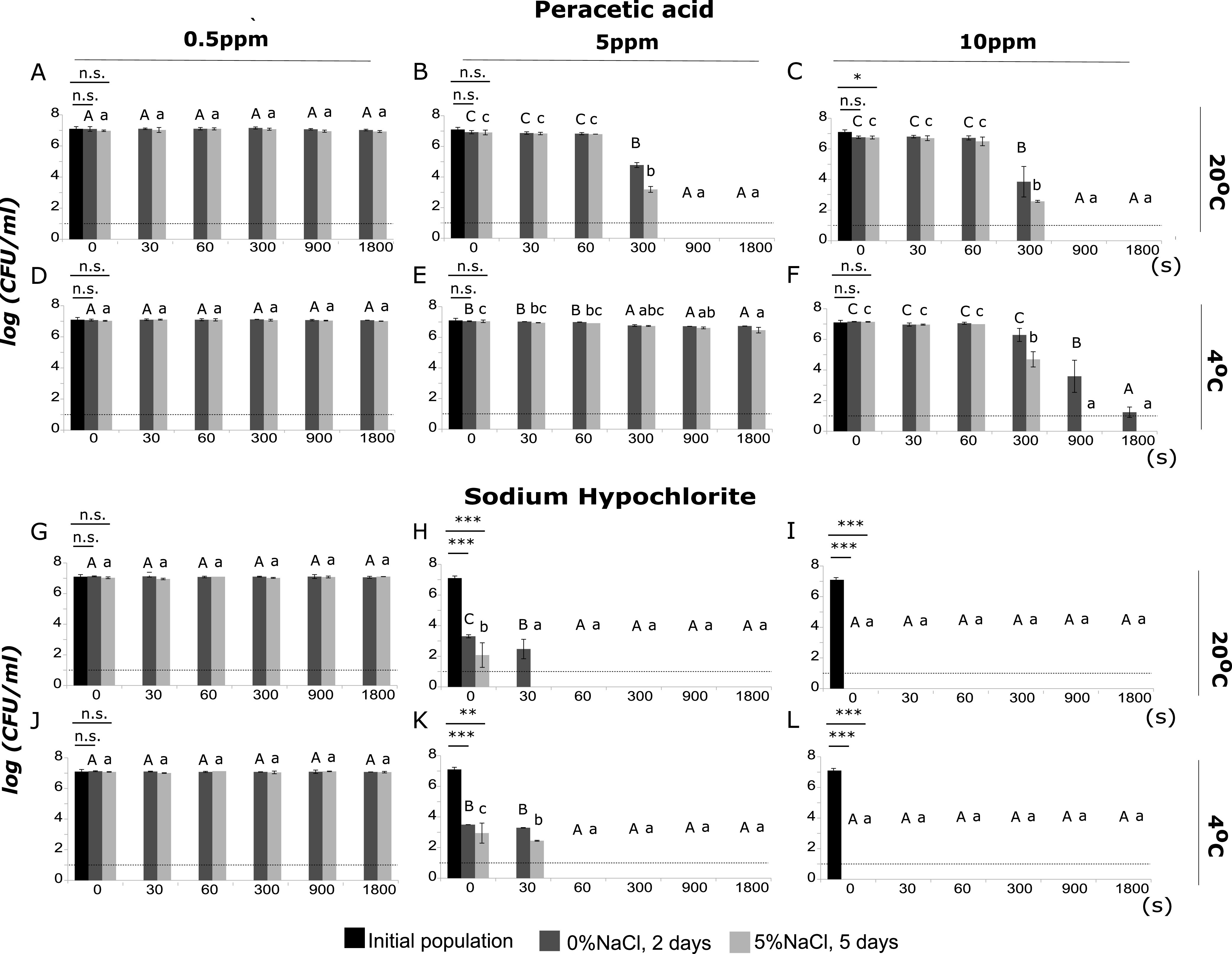
Populations (log CFU/ml) of L. monocytogenes, strain Scott A, after incubation for 0, 30, 60, 300, 900, or 1,800 s under the following conditions: (A) PAA, 0.5 ppm, at 20°C, (B) PAA, 5 ppm, at 20°C, (C) PAA, 10 ppm, at 20°C, (D) PAA, 0.5 ppm, at 4°C, (E) PAA, 5 ppm, at 4°C, (F) PAA, 10 ppm, at 4°C, (G) SH, 0.5 ppm, at 20°C, (H) SH, 5 ppm, at 20°C, (I) SH, 10 ppm, at 20°C, (J) SH, 0.5 ppm, at 4°C, (K) SH, 5 ppm, at 4°C, and (L) SH, 10 ppm, at 4°C. Total culturable (dark gray bars) and noninjured (light gray bars) population levels were measured after incubation for 2 days and 5 days at 37°C on TSAYE supplemented with 0% and 5% NaCl, respectively. Bars represent mean values ± SD from 3 independent biological replicates. Statistically significant differences (*P* < 0.05) are indicated with different uppercase (TSAYE) and lowercase (TSAYE+5%NaCl) letters, respectively. The dotted line represents the limit of detection (LOD). *, *P* < 0.1; **, *P* < 0.05; ***, *P* < 0.01; n.s., not significant.

**FIG 3:**
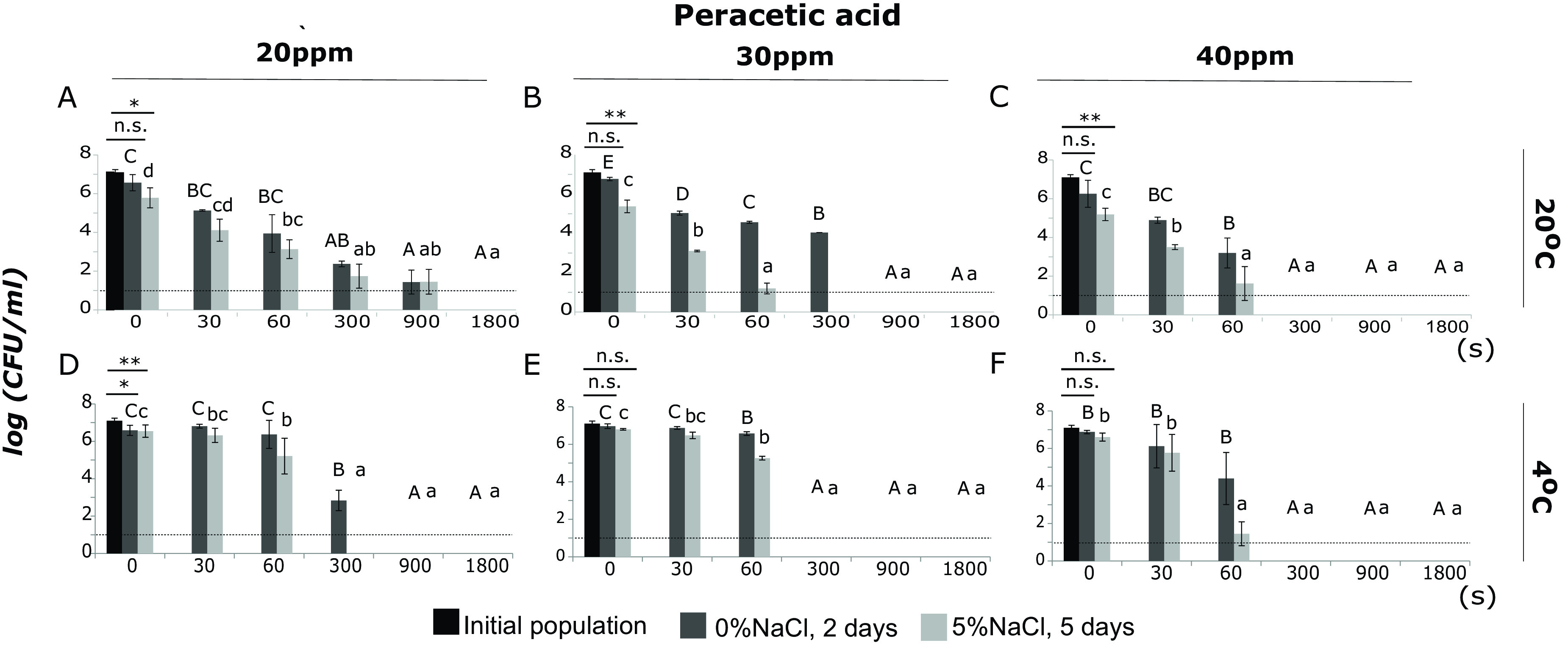
Populations (log CFU/ml) of L. monocytogenes, strain Scott A, after incubation for 0, 30, 60, 300, 900, or 1,800 s under the following conditions: (A) PAA, 20 ppm, at 20°C, (B) PAA, 30 ppm, at 20°C, (C) PAA, 40 ppm, at 20°C, (D) PAA, 20 ppm, at 4°C, (E) PAA, 30 ppm, at 4°C, and (F) PAA, 40 ppm, at 4°C. Total culturable (dark gray bars) and noninjured (light gray bars) population levels were measured after incubation for 2 days and 5 days at 37°C on TSAYE supplemented with 0% and 5% NaCl, respectively. Bars represent mean values ± SD from 3 independent biological replicates. Statistically significant differences (*P* < 0.05) are indicated with different uppercase (TSAYE) and lowercase (TSAYE+5%NaCl) letters, respectively. The dotted line represents the limit of detection (LOD). *, *P* < 0.1; **, *P* < 0.05; n.s., not significant.

Exposure to PAA resulted in the formation of sublethally injured cells and affected the survival of L. monocytogenes, both at 20°C and 4°C. However, cells treated with 0.5 ppm PAA at 20°C or 4°C retained their culturability and remained at levels of up to 7 log CFU/ml after 30 min of incubation (Fig. 2A and D). Increasing the stress intensity from 0.5 ppm to 5 ppm PAA at 20°C resulted in significant decreases of the total culturable population and the population enumerated on TSAYE+5%NaCl, by 2.1 and 3.7 log units, respectively, after 5 min (*P* < 0.05) (Fig. 2B). At the same time, the level of injury was up to 1.6 log units (Fig. 2B). At 4°C, the total culturable population remained between 6.7 and 7.0 log CFU/ml throughout the time of exposure (Fig. 2E). Exposure to 20 ppm PAA at 20°C resulted in a significant reduction of the total culturable population after 15 min of exposure, by up to 5.13 log (*P* < 0.05) (Fig. 3A). The population enumerated on TSAYE+5%NaCl was significantly lower than the initial population (untreated cells) at time zero of exposure to 30 and 40 ppm PAA at 20°C (*P*< 0.05) (Fig. 3B and C). Incubation for 1 min at 20°C in the presence of 30 ppm PAA showed a significant reduction of noninjured cells (Fig. 3B). The same pattern was observed after incubation for 1 min at 20°C in 40 ppm PAA (Fig. 3C). At 4°C, the total culturable population was not significantly different from the initial population at time zero after exposure to 30 and 40 ppm PAA (Fig. 3E and F). Furthermore, the total culturable population showed significant reductions after 30 s and 1 min of exposure to 30 ppm PAA at 4°C (Fig. 3E). In parallel, after 1 min of incubation under the same conditions, the populations of L. monocytogenes (total and noninjured) were below the LOD (1 log CFU/ml) (Fig. 3E). Increasing the stress intensity from 30 to 40 ppm PAA at 4°C resulted in a similar level of the total culturable population until 1 min of exposure and significant reductions after 5 min (below the LOD) (Fig. 3F).

Comparisons of the total mean populations of L. monocytogenes after incubation for 0 s, 30 s, and 1 min at 20°C revealed two comparable inactivation patterns, one for 0.5, 5, and 10 ppm PAA and another for 20, 30, and 40 ppm (Table S3). The total culturable population levels of the first group were significantly higher. Exposure to 5, 10, 20, 30, and 40 ppm PAA at 20°C for 5, 15, and 30 min resulted in significant reductions of the total culturable populations compared to exposure to 0.5 ppm. More specifically, at 30 min of exposure, the total culturable population was below the LOD (1 log CFU/ml). The same pattern was observed for the mean populations of noninjured cells after incubation for 15 and 30 min at 20°C. At 4°C, significant reductions of the total culturable populations were detected after exposure to 20 and 30 ppm PAA for 5 min. Increasing the time of exposure from 5 to 15 min resulted in significant decreases of the total culturable populations of L. monocytogenes exposed to 10 and 20 ppm PAA. Finally, after 30 min of exposure to 5 and 10 ppm PAA, reductions of the total culturable populations were observed, indicating a progressive inactivation pattern that depended on the time of exposure. The populations on TSAYE+5%NaCl showed significant reductions after exposure to 5 and 10 ppm PAA for 15 and 30 min at 4°C.

Exposure to SH at 5, 10, 20, 30, and 40 ppm significantly affected the inactivation of L. monocytogenes populations (total culturable and noninjured populations). At time zero, after exposure to 5 ppm SH at 20°C, the total culturable population and the population enumerated on TSAYE+5%NaCl decreased by 3.8 and 5.0 log units, respectively, compared to the initial population level (untreated cells) (*P* < 0.01) (Fig. 2H). Incubation for 30 s under the same conditions resulted in an approximately 2-log CFU/ml reduction of noninjured cells (Fig. 2H). However, after 30 s of exposure, the L. monocytogenes population was below the LOD (1 log CFU/ml) (Fig. 2H). Exposure to 5 ppm SH at 4°C had a significant impact on the total culturable and noninjured populations at time zero (*P* < 0.01) (Fig. 2K). Additionally, incubation for 30 s showed detectable numbers of injured cells, i.e., 0.8 log (Fig. 2K). Plate counting showed sharp reductions in bacterial counts following treatment by SH at 10 ppm (Fig. 2I and L) and 20, 30, and 40 ppm (data not shown) for 30 min at 20°C and 4°C, indicating that L. monocytogenes is sensitive to chlorine. Under the aforementioned conditions, the population levels were below the LOD.

The total mean population and the population on TSAYE+5%NaCl differed between 0.5 and 5 ppm SH at 20°C at time zero, showing significant reductions in the population levels of L. monocytogenes. Furthermore, comparison between the initial and total culturable populations after exposure to 5 ppm SH at 20°C and 4°C at time zero revealed a highly significant decrease in total culturable population levels (*P* < 0.01). The same pattern was observed between 10, 20, 30, and 40 ppm SH, where the populations on TSAYE and TSAYE+5%NaCl were below the LOD. After incubation for 1 min at 20°C and 4°C, total culturable and noninjured populations were detected only in 0.5 ppm SH, indicating the detrimental effect of SH on L. monocytogenes’ survival.

### Phenotypic change of colonies.

**FIG 4:**
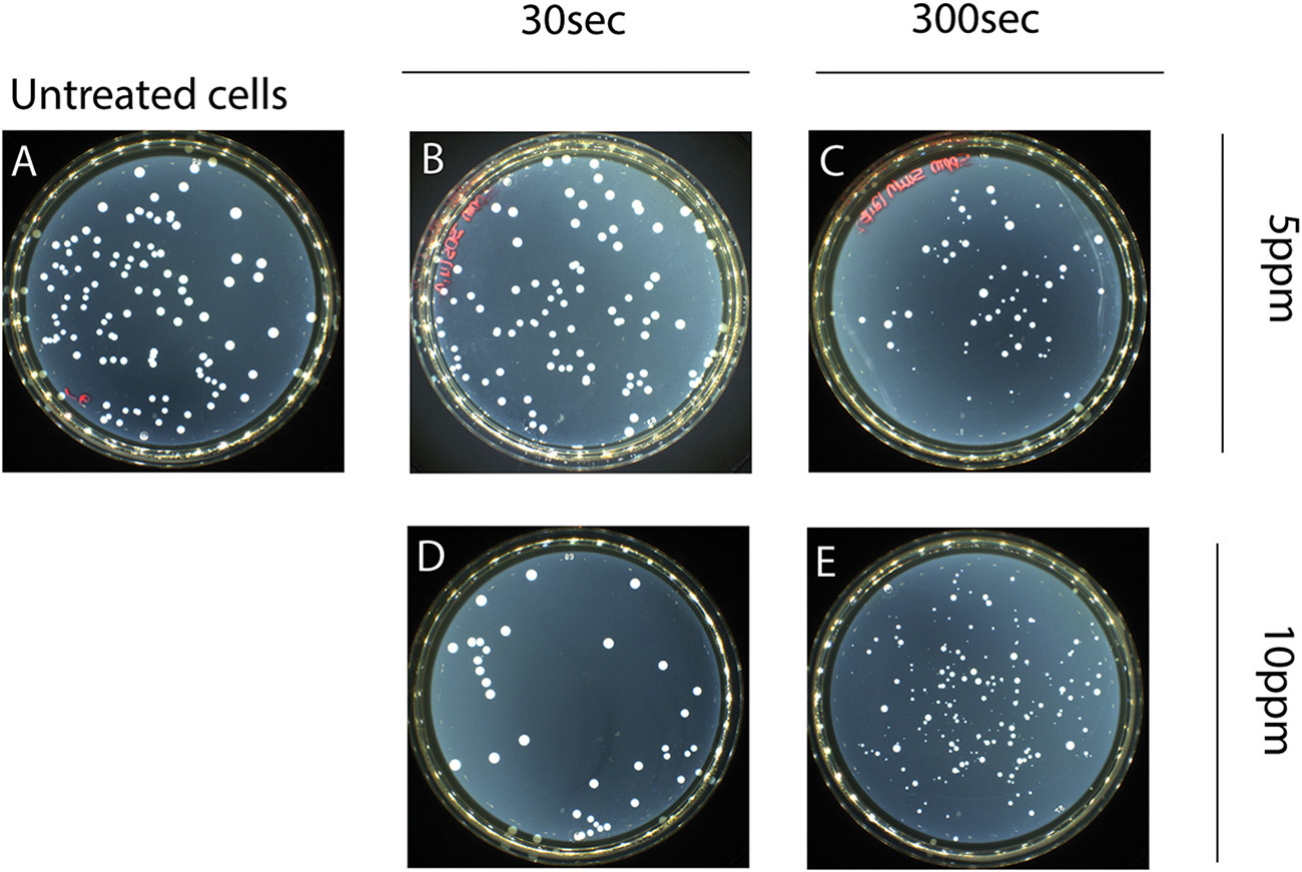
Alterations in colony morphotype after exposure of L. monocytogenes to PAA at 5 ppm for 30 s (B) or 300 s (C) and at 10 ppm for 30 s (D) or 300 s (E) at 20°C. The total population on TSAYE showed colony morphology variations. Based on visual inspection, two subpopulations were observed, the untreated cells’ colony phenotype (A) and small colony variants (SCVs), indicating phenotype heterogeneity.

**FIG 5:**
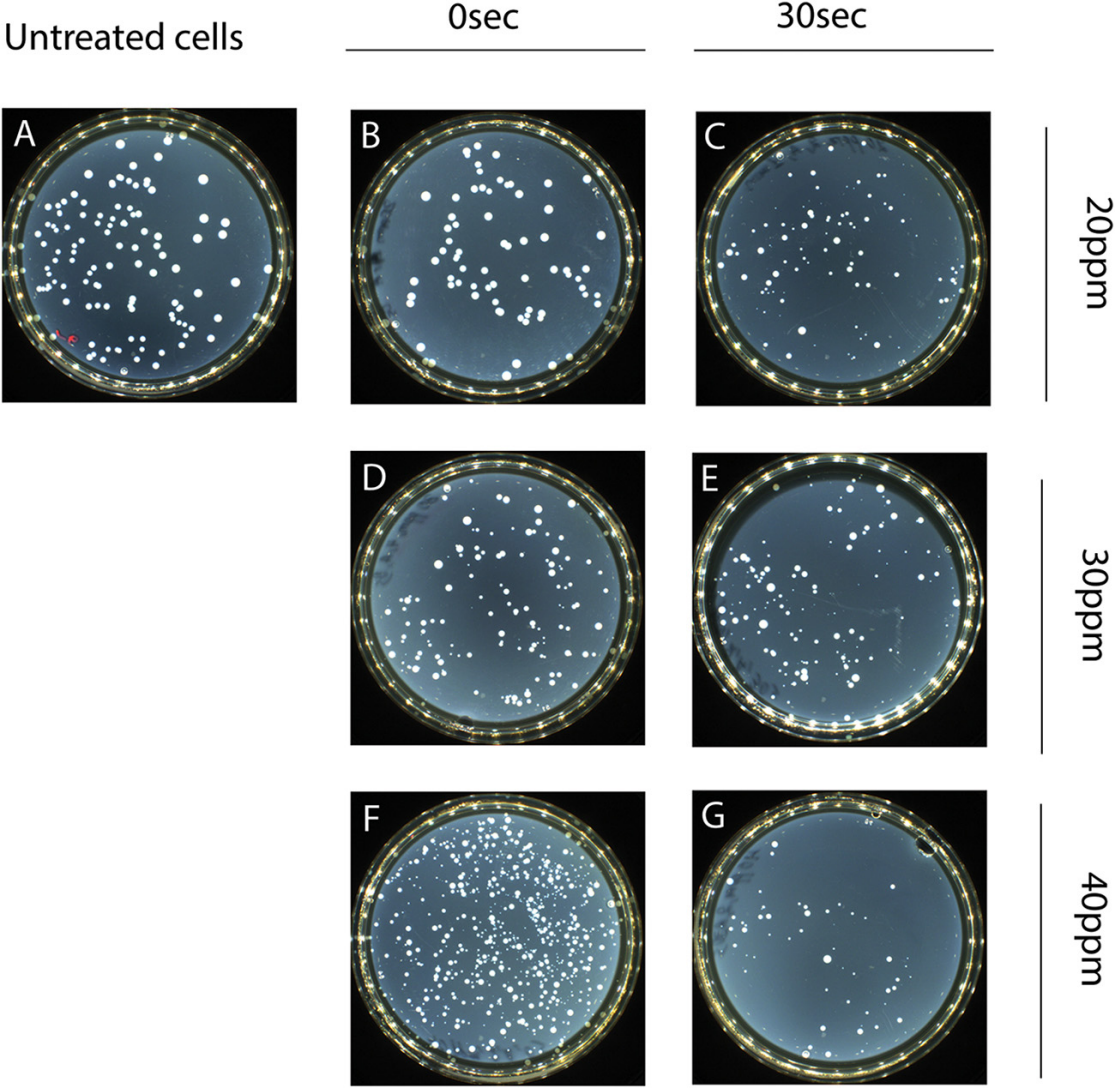
Alterations in colony morphotypes after exposure of L. monocytogenes to PAA at 20 ppm for 30 s (B) or 300 s (C), at 30 ppm for 30 s (D) or 300 s (E), and at 40 ppm for 30 s (F) or 300 s (G) at 20°C. The total population on TSAYE showed colony morphology variations. Based on visual inspection, two subpopulations were observed, the untreated cells’ colony phenotype (A) and small colony variants (SCVs), indicating phenotype heterogeneity.

Acetic acid- and hydrochloric acid-treated cells did not show colony morphology variants in the total culturable population (CFU on TSAYE). Regarding the effect of PAA on the L. monocytogenes colony morphotype, we observed morphology variations in the total culturable population colonies (CFU on TSAYE), depending on the stress intensity and time of exposure. Two subpopulations (morphotypes) were detected under macroscopic colony observation, one with the phenotype of untreated cells and one comprising SCVs. SCVs were first observed after 5 min of exposure to 5 ppm PAA at 20°C (Fig. 4C). To further confirm our preliminary observations and hypothesis for the existence of subpopulations, possibly with different recovery growth kinetics, we observed the colony morphotypes after all treatments following 2 days of incubation at 37°C. It was clear that as the stress intensity increased from 5 to 40 ppm PAA, SCVs were detected at earlier times of exposure, indicating that phenotype heterogeneity may be triggered by external factors (Fig. 5F and G). Notably, SCVs were present on TSAYE shortly (10 s) after the initiation of exposure to 30 and 40 ppm PAA at 20°C (Fig. 5D and F).

### Induction of VBNC state.

Evaluation of the effects of AA, HCl, PAA, and SH on sublethal injury and inactivation of L. monocytogenes, as well as the observation of SCVs after treatment with different concentrations of PAA, allowed us to select which conditions may induce the VBNC state. As a result, the survival of L. monocytogenes cells was evaluated comparatively (plate counting and fluorescence microscopy) after exposure to the disinfectant PAA (20, 30, and 40 ppm) at 20°C. In order to distinguish metabolically active, injured, and dead cells, cells were stained with a mixture of CFDA and propidium iodide (PI). Quantification of viable (CFDA-positive [CFDA^+^]/PI-negative [PI^−^]), dead (CFDA-negative [CFDA^−^]/PI-positive [PI^+^]), injured (CFDA^+^/PI^+^), and unstained cells was performed to capture the distribution of populations of different physiological states at the single-cell level. In parallel, plate counting was used for the evaluation of the surviving populations (total, noninjured, and injured).

**FIG 6:**
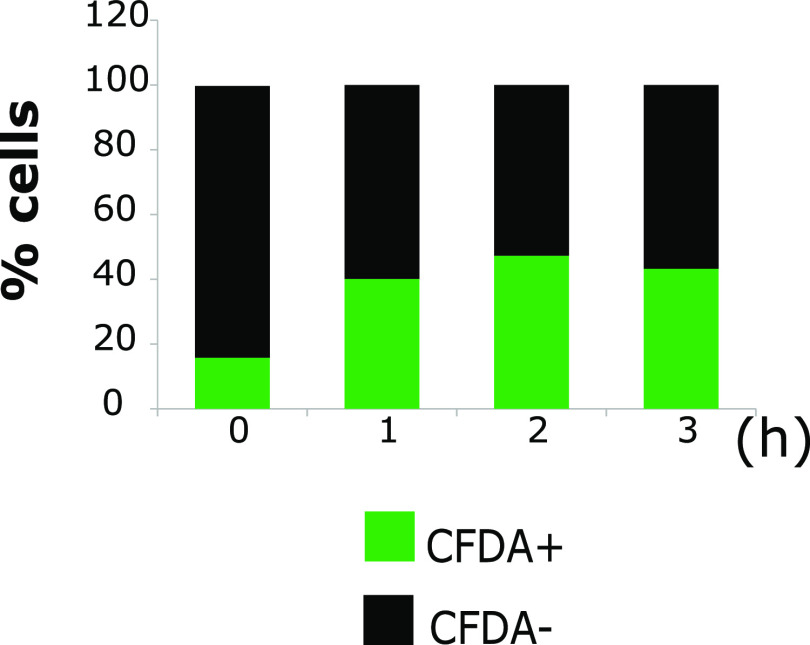
Enumeration results (%) for untreated cells (positive control) exhibiting fluorescence (CFDA^+^, green, or CFDA^−^, black), considering several images of different fields of view (FOV) at each time point (0 h, 5 FOV; 1 h, 4 FOV; 2 h, 4 FOV; and 3 h, 5 FOV).

Preliminary experiments were performed in order to evaluate and standardize our staining procedures. As shown by the results in Fig. 6, following 1, 2, and 3 h of incubation in Ringer’s solution at 20°C, the percentages of CFDA^+^ cells were 40.00%, 47.27%, and 43.22%, respectively. We assume that cells remaining unstained was due to their inability to rapidly adjust to the change of solution (TSBYE to Ringer’s solution) and extracellular pH, i.e., from TSBYE with pH 5.1 ± 0.01 (mean ± SD) to Ringer’s solution with pH 7.5 ± 0.5. For confirming negative controls (dead cells), the above-mentioned cultures were exposed to 70% ethanol for 30 min at room temperature. The ethanol-treated cells were stained with PI. The percentage of PI^+^ cells was approximately 99.8% (data not shown), indicating that cells with damaged membranes (considered dead) were successfully stained. Double staining with CFDA/PI offered the best and most versatile indication of both cell metabolic activity and membrane integrity.

**FIG 7:**
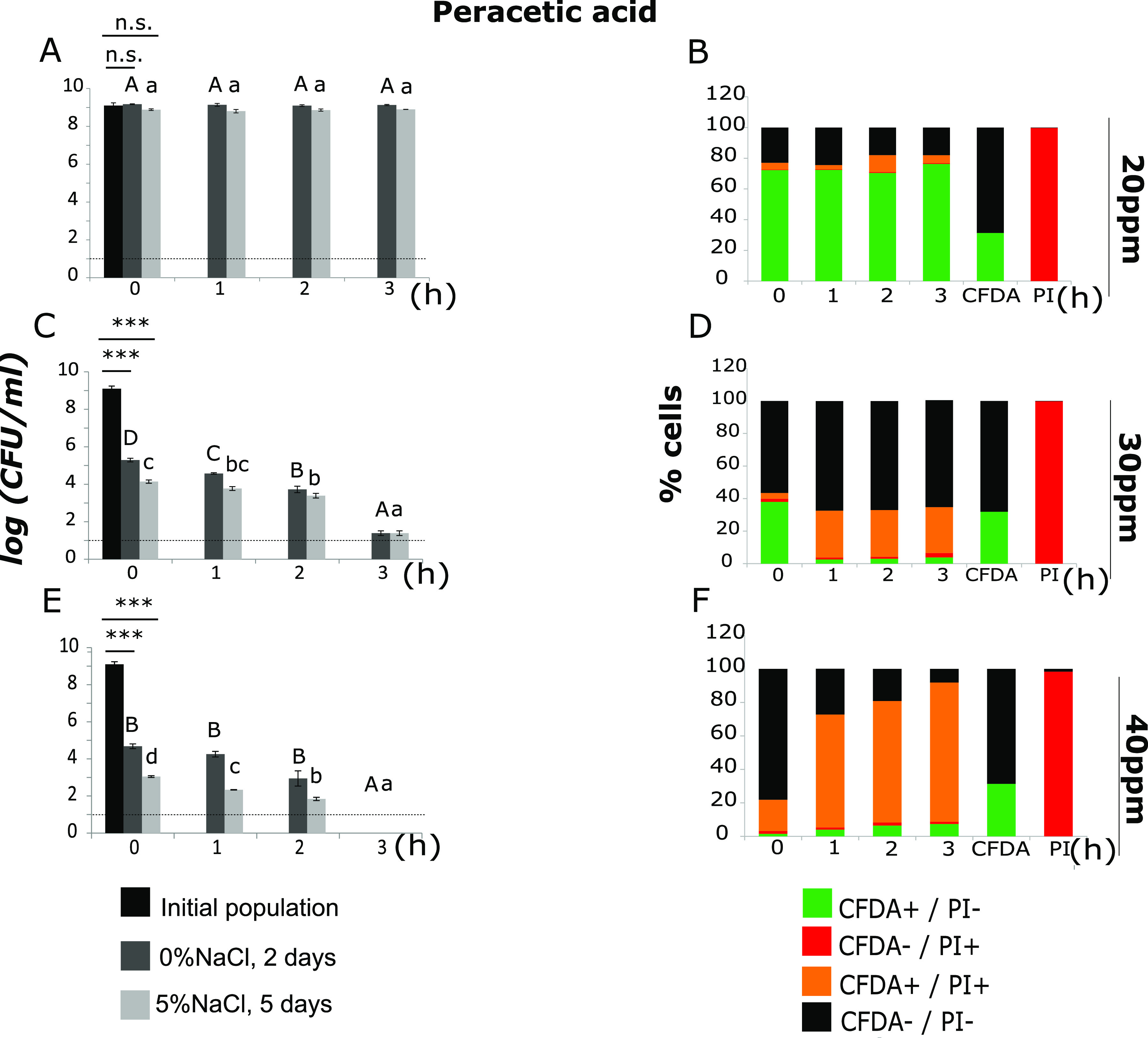
Populations (log CFU/ml) of L. monocytogenes, strain Scott A, after incubation for 0, 1, 2, or 3 h at 20°C in Ringer’s solution with 20 ppm (A), 30 ppm (C), or 40 ppm (E) PAA. Total culturable (dark gray bars) and noninjured (light gray bars) population levels were measured after incubation for 2 and 5 days at 37°C on TSAYE supplemented with 0% and 5% NaCl, respectively. Bars represent mean values ± SD from one experiment with six independent samples (*n* = 6). Statistically significant differences (*P* < 0.05) are indicated with different uppercase and lowercase letters for TSAYE and TSAYE+5%NaCl, respectively. The dotted line represents the limit of detection (LOD). ***, *P* < 0.01; n.s., not significant. Each time point relates to several images acquired at different fields of view (FOV) of the same sample. Enumeration results (%) for cells exhibiting fluorescence (CFDA^+^/PI^−^, green; CFDA^−^/PI^+^, red; CFDA^+^/PI^+^, orange; or CFDA^−^/PI^−^, black), considering several images of different fields of view (FOV) at each time point after treatment with PAA at 20 ppm (0 h, 5 FOV; 1 h, 4 FOV; 2 h, 5 FOV; 3 h, 5 FOV) (B), 30 ppm (0 h, 4 FOV; 1 h, 5 FOV; 2 h, 5 FOV; 3 h, 5 FOV) (D), and 40 ppm (0 h, 4 FOV; 1 h, 5 FOV; 2 h, 5 FOV; 3 h, 5 FOV) (F).

Incubation for 3 h in Ringer’s solution with 20 ppm PAA at 20°C had a sublethal effect on L. monocytogenes cells (Fig. 7A). More specifically, when 10^9^ CFU/ml were exposed to 20 ppm PAA, the cells retained their culturability and remained at the same initial population level for 3 h. Also, a comparison of the total culturable and noninjured populations to the initial population did not show any statistically significant decrease. As far as the fluorescence results are concerned, the percentages of CFDA^+^/PI^−^ cells ranged between 70.47 and 76.33% during the exposure, confirming the existence of cellular metabolic activity and membrane integrity (Fig. 7B). However, the percentages of double-stained cells (CFDA^+^/PI^+^) increased after 2 h of incubation, indicating the existence of injured cells also (Fig. 7B). This fraction of injured cells evaded detection using the plate counting method, due to the fact that plate counting detects the total population trend above a limit of detection (LOD) (1 log CFU/ml).

Increasing the stress intensity from 20 to 30 ppm PAA resulted in significant reductions of L. monocytogenes total culturable and noninjured populations at time zero, by 3.8 and 4.9 log units, respectively, compared to untreated cells (*P* < 0.01) (Fig. 7C). At the same time, the percentage of CFDA^+^/PI^−^ cells was 38.01%, while the percentages of CFDA^−^/PI^+^ and CFDA^+^/PI^+^ cells were 1.91% and 3.56%, respectively (Fig. 7D). The total population level decreased after incubation for 3 h in 30 ppm PAA, reaching 1.59 log CFU/ml (Fig. 7C). The population of noninjured cells showed progressive inactivation and decreased significantly after 3 h of exposure. At time zero of exposure, the percentage of CFDA^+^/PI^+^ cells increased and was stabilized to approximately 28%. The percentages of CFDA^+^/PI^−^ cells were between 2.87% and 3.83%, while the percentage of CFDA^−^/PI^+^ cells after 3 h of exposure to 30 ppm PAA was 2.69%.

The total culturable and noninjured populations at time zero were significantly decreased after exposure to 40 ppm PAA at 20°C compared to the initial populations (*P* < 0.01) (Fig. 7E). Incubation for 1 and 2 h did not affect the total population levels, while after 3 h, the population*s* (total and noninjured) were below the LOD (1 log CFU/ml). The population enumerated on TSAYE+5%NaCl decreased significantly over time. The proportion of injured cells was 1.63 log CFU/ml at time zero, whereas after incubation for 1 and 2 h, the proportions of sublethally injured cells were 1.92 and 1.10 log CFU/ml, respectively. The percentage of double-stained cells (CFDA^+^/PI^+^) increased progressively during the exposure, indicating sublethal injury at the single-cell level. Similarly, the percentage of cells exhibiting green fluorescence (metabolic activity and membrane integrity) was 1.51% at time zero and increased to 7.36% after 3 h of exposure to 40 ppm PAA (Fig. 7F). Importantly, when L. monocytogenes Scott A strain was exposed to Ringer’s solution with 40 ppm PAA at 20°C for 3 h, the whole population was considered nonculturable (using plate counting); however, at the single-cell level, there were cells exhibiting green fluorescence (viable). These results corroborate the induction of the VBNC state.

## DISCUSSION

The existence of different forms of dormant states (persisters and VBNC state) in nonsporulating microbes has previously been described as a part of a shared “dormancy continuum” (38). The main objective of this research was to examine, at the single-cell and population levels, sublethal injury in L. monocytogenes, using plate counting to evaluate alterations in colony morphotype indicating persistence and to distinguish physiologically heterogeneous fractions of cells (culturable, injured, VBNC, and dead). In order to investigate the sublethal injury phenotype and VBNC state, we used plate counting and fluorescence microscopy in parallel. The advantages of the combined methods are that they increase the resolution of the VBNC or injury assessment and also overcome problems related to the enumeration limit of a single enumeration method, which in our case was plate counting. In the same context, they are useful in order to better define the safety concerns arising from sublethal injury and the VBNC state.

### Sublethal stresses may induce injury.

According to our results, at the population level, induction of sublethal injury was more intense after treatment with weak acid (AA) at the temperatures tested (20°C and 4°C). Acetic acid, as a weak organic acid, is protonated at low pH (pH < pK_a_) and therefore uncharged and more lipophilic, and thus, it is able to permeate the lipid bilayer, trigger cytoplasmic acidification, and collapse proton gradients (39). On the other hand, HCl (a strong inorganic acid) exists as a dissociated form under most physiological pH values (40). Acetic acid has a higher pK_a_ (4.76 at 25°C) than HCl and as a result is expected to accumulate in cells at higher concentrations, causing acidification of the cytoplasm and an additional toxic effect (41). Another finding, in agreement with the results reported by Zhang et al. (42), is that the population levels of L. monocytogenes are higher after exposure to AA and HCl at 4°C than at 20°C, indicating that acid injury is affected by the incubation temperature. Ahamad and Marth observed that acid-treated cells of L. monocytogenes needed less time for population decrease or injury after incubation at 35°C than at 13°C (43). Similarly, Siderakou et al. reported that the injury levels of L. monocytogenes (Scott A strain) gradually increased within the first 8 h of exposure to Ringer’s solution at pH 3, adjusted with lactic acid, at 20°C, with the same inactivation pattern being observed at 4°C (44). Sibanda and Buys have also demonstrated extensive cell membrane damage and a high degree of injury in *Listeria* cells as a result of exposure to lactic acid (45).

Our results concerning the exposure to disinfectants (PAA and SH) indicate that SH has a significant effect on the survival of L. monocytogenes populations (total and noninjured), showing population levels below the LOD (1 log CFU/ml) after a short exposure to 10 ppm (20°C and 4°C). It has also been reported that planktonic cells of L. monocytogenes Scott A strain showed high sensitivity to hypochlorite, being totally eliminated after 30 s of exposure to 10 and 20 ppm (46). The same inactivation pattern was observed after incubation in the presence of 5 ppm hypochlorite. Similarly, Gu and coworkers observed that after 30 s in sterile water with 0.5 ppm free chlorine, L. monocytogenes became undetectable by selective plating (34). SH belongs to the chlorine-releasing agents (CRAs) and is widely used as a disinfectant or a bleaching agent (47). In aqueous solution, it exists in three different forms, Cl_2_, hypochlorous acid (HOCl), and OCl^-^, which are called free available chlorine or free residual chlorine (48). The form of chlorine predominantly depends on the pH value of the SH solution. Between pH 4.0 and 7.0, HOCl predominates (47), with antimicrobial activity similar to that of undissociated organic acids. The mechanisms of germicidal action are different between HOCl and -OCl, as HOCl can penetrate the lipid bilayer by passive diffusion, attacking the microbial cells both from the outside and the inside, while -OCl cannot, exerting its oxidizing action only from the outside (48).

PAA belongs to the peroxygens (48), and as a selective oxidizing agent, it has the ability to remove electrons, preferring electron-rich moieties like aromatic rings and double and triple bonds (49). Also, it can oxidize sulfhydryl (-SH) and disulfide (S-S) bonds in proteins and enzymes (50). In our experiments, PAA had a milder effect than SH, inducing sublethal injury but allowing cellular recovery following 1 min of exposure to 40 ppm. This is consistent with the observations by Gu et al., who reported that incubation for 30 s in 30 ppm PAA resulted in undetectable population levels of L. monocytogenes by selective plating (34).

### Supporting evidence for phenotypic switching.

Macroscopic observation of PAA-treated cells strongly suggested the presence of variations in the morphology of total population colonies (CFU on TSAYE). SCVs were first detected following 5 min of exposure to 5 ppm PAA at 20°C. The presence of this colony morphotype seemed to depend on the intensity of the stress and the time of exposure. As L. monocytogenes showed tolerance after exposure to PAA, we propose that SCVs may be a transient phenotypic switch that possibly indicates a persistent subpopulation. As reported by Keren and colleagues (51), persisters are specialized survivor cells. This physiological state is a form of dormancy, closely related to the VBNC state (38), that may be induced independently of the use of antibiotics. Phenotypic switching is closely related to persistence (29) and plays a beneficial role in the evolution process, allowing bacteria to survive against changes in external environmental factors (29, 52). This phenomenon induces microbial heterogeneity by creating phenotype variants that are of high “fitness” and, thus, better adapted to the new environment (53). Our observations indicate the existence of two distinct subpopulations with different colony morphotypes after exposure to PAA, a phenomenon which may be induced by external factors (“responsive switching”) in order to strengthen the ability of L. monocytogenes to survive.

### Induction of the VBNC state.

Based on our sublethal injury results, we proceeded in the evaluation of the potential induction of the VBNC state after treatment with PAA. PAA is an oxidizing agent, commonly used in food-processing environments, which, as described above, may induce sublethal injury and persistence in L. monocytogenes. For these reasons, we assessed the metabolic activity and potential loss of culturability of L. monocytogenes after exposure to 20, 30, and 40 ppm PAA for 3 h at 20°C, using fluorescence microscopy and plate counting in parallel. Each sampling point was related to several images (4 or 5) acquired at different fields of view (FOV) of the same sample, indicating metabolic activity or death. To succeed in this, we exposed 10^9^ CFU/ml L. monocytogenes to stress, i.e., a markedly higher population than the one used in plate count assays, to enable detectable single cells within the microscopy optical field.

In order to distinguish different fractions of cells (culturable, injured, VBNC, and dead), we used a mixture of CFDA and PI. It was recently acknowledged that among five methods for enumerating L. monocytogenes in the VNBC state, i.e., (i) LIVE/DEAD BacLight staining, (ii) ethidium monoazide and propidium monoazide staining followed by real-time PCR (EMA- and PMA-PCR), (iii) direct viable count (DVC), (iv) 5-cyano-2,3-ditolyl tetrazolium chloride-40, 6-diamidino-2-phenylindole (CTC-DAPI) double staining, and (v) CFDA staining, LIVE/DEAD BacLight staining and CFDA-DVC staining currently appear to be the most accurate (37). In the positive control (untreated cells stained with CFDA), there was a percentage of cells exhibiting no fluorescence. CFDA is a pH-sensitive fluorescent probe, used to measure the pH intracellular (pH_in_) and to indicate cell viability (54). Fluorescence occurs when the nonfluorescent CFDA permeates the cell membrane and then is converted by cytosolic esterases to the highly fluorescent carboxyfluorescein (CF), which because it is negatively charged, remains inside the cell (55). Bunthof and colleagues reported that stained cells retained CF well when not energized but extruded the probe rapidly upon the addition of lactose (56). This indicates that the extrusion is most likely mediated by an ATP-driven transport system (56). This may further explain the fact that our positive control also contained unstained cells. The activated cultures were incubated for 18 h at 30°C in TSBYE, which contains 2.5 g/liter glucose. We assume that the detectable unstained subpopulation could not adjust rapidly to the change of solution and pH, i.e., from TSBYE, pH 5.1 ± 0.01, to Ringer’s solution, pH 7.5 ± 0.5, and thus, did not show any fluorescence. Obviously, this could justify the fact that the percentage of unstained cells decreased over time, from 83.9% to 56.78%. On the other hand, ethanol-treated cells were well stained with PI (99.8%), indicating that the dye successfully passes the damaged membrane and binds to DNA.

Exposure to 30 and 40 ppm PAA showed that a large proportion of bacterial cells were injured. There was a sharp, logarithmic reduction in population levels (total and noninjured), as well as a significant percentage of double-stained cells (CFDA^+^/PI^+^), indicating metabolic activity and membrane damage. This progressive increase in injured cells was followed by a fraction of nonculturable but viable cells after 3 h of exposure to 40 ppm PAA at 20°C. These results confirm that PAA induces the VBNC state in L. monocytogenes. Furthermore, the fluorescence results showed that as the stress intensity increased, the percentage of unstained cells was mainly replaced by CFDA^+^/PI^+^ cells. We suggest that those unstained cells represent a fraction of cells that is in the initial stages of dormancy, where cells exit viability but retain their membrane integrity. Increasing the stress intensity leads cells to sublethal injury, persistence, and induction of the VBNC state. In agreement with our findings, Gu et al. reported the presence of L. monocytogenes VBNC cells after treatment by 30 and 50 ppm PAA (34). In fact, it is not evident whether VBNC cells remain avirulent or infectious, but a public health threat theoretically exists, because the virulence armory is retained, and thus, infection can be initiated upon resuscitation, e.g., in the host (12). Since in this study, we showed that PAA treatment induces a heterogenous mixture of cells containing a fraction in the VBNC state, it would be interesting to separate each subpopulation to further evaluate its phenotypic characteristics.

### Conclusions.

In the present study, we confirmed that sublethal injury is an initial stage of dormancy in L. monocytogenes that is followed by persistence and the VBNC state. Our results showed that PAA may induce persistence and the VBNC state in L. monocytogenes, indicating that nonculturability may provide false-positive results regarding the microbial load of a product. Understanding dormancy kinetics will highlight the potent dangers of consuming food contaminated with dormant cells. Also, it will help develop effective ways to prevent foodborne illnesses and support decision-making at the national level.

## MATERIALS AND METHODS

### Bacterial strains and preparation of inoculum.

Listeria monocytogenes, Scott A strain, a clinical isolate (serotype 4b) from a foodborne listeriosis outbreak (57), was used in this study. Stock cultures were stored at −20°C in tryptic soy broth (TSB; Oxoid) supplemented with 0.6% yeast extract (Lab M) (TSBYE) and 20% glycerol. Starting from the stock cultures, colony cultures were maintained on plates of tryptic soy agar with 0.6% yeast extract (TSAYE) under refrigeration at 4°C.

A single colony from TSAYE stock culture was transferred by loop into 10 ml TSBYE and incubated for 24 h at 30°C. Next, 0.1 ml of the 24-h culture was transferred into 10 ml TSBYE and incubated for 18 h at 30°C to obtain stationary-growth-phase cells (Fig. S1). The cells were washed twice in 1/4-strength Ringer’s solution (Lab M), to maintain the osmotic balance of the bacteria, and harvested by centrifugation (2,434 × *g* for 10 min at 4°C). The initial population was determined by plating 0.1 ml of the appropriate dilution on a TSAYE plate and incubating for 48 h at 37°C. The initial population of the Scott A strain was established at approximately 10^9^ CFU/ml.

### Preparation of stress solutions.

Two types of stress conditions were used in this study to induce sublethal cell injury: acid stress, using AA or HCl, and disinfectant stress, by exposure to PAA or SH. Cells were exposed to the stress agents at two temperatures, namely, 4°C (refrigeration) or 20°C (ambient conditions in food-processing environments). The exposure conditions were chosen to represent the potential sublethal stresses encountered by L. monocytogenes in foods and food-processing environments. Stress solutions were prepared first and equilibrated for at least 20 min at the two temperatures, 4°C or 20°C.

### Preparation of acid stress solutions.

Acetic acid solution at 1 M (stock solution) and hydrochloric acid solution at 3 N (stock solution) were prepared in order to adjust the pH to the targeted acidity. Stock solution was gradually added to 50 ml sterile Ringer’s solution under aseptic conditions. pH values were recorded until the target pHs of 3.0, 2.7, and 2.5 were achieved.

### Preparation of PAA stress solution.

To prepare a stock solution of peracetic acid (15% pure; AppliChem), 50 μl was dissolved aseptically in 50 ml of sterile Ringer’s solution. The concentration of PAA was 150 ppm. An appropriate quantity of the stock solution was added to 120 ml of sterile Ringer’s solution until a final concentration of 0.5 (pH 6.93), 5 (pH 4.96), 10 (pH 4.50), 20 (pH 4.25), 30 (pH 4.10), or 40 (pH 3.98) ppm was reached (working solution). As PAA solution is unstable, its concentration was monitored throughout the exposure at 4°C or 20°C, using a semiquantitative colorimetric method (peracetic acid test strips; Merck).

### Preparation of SH stress solution.

Sodium hypochlorite (12% active chlorine; Merck) stock solution (120 ppm) was prepared by dissolving 50 μl in 50 ml of sterile Ringer’s solution. Then, working SH solutions were prepared from the stock solution to final concentrations of 0.5 (pH 7.83), 5 (pH 8.68), 10 (pH 8.91), 20 (pH 9.22), 30 (pH 9.40), and 40 (pH 9.54) ppm. The stability of the SH working solutions was monitored during exposure at 4°C or 20°C, using the *N*,*N*-diethyl-*p*-phenylenediamine (DPD) method via an electronic Scuba II pool tester instrument, and the magnitude of the free chlorine concentration was measured semiquantitatively (chlorine test strips; Merck).

### Exposure to stress conditions.

The activated stationary-phase cultures (10^9^ CFU/ml for Scott A) were centrifuged twice (2,434 × *g* for 10 min at 4°C, to avoid additional injury caused by the increase in temperature during centrifugation) and resuspended in 10 ml Ringer’s solution. Next, 100 μl of cell culture was transferred into 10 ml of the chosen stress solution (approximately 10^7^ CFU/ml). The exposure time and sampling time points varied depending on the type of stress and the time needed for complete inactivation. In order to assess the metabolic activity with fluorescence microscopy, the activated cultures (approximately 10^9^ CFU/ml, Scott A strain), after being washed once (2,434 × *g* for 10 min at 4°C), were resuspended in 3 ml of solution containing the stress agent.

### Evaluation of sublethal injury by plate counting.

To differentiate the injured subpopulation from the total culturable population, growth in TSAYE supplemented with 5% NaCl (wt/vol) (AppliChem) was compared to growth in TSAYE with 0.5% NaCl. This method relies on the inability of injured cells to grow on selective media (58, 59). A concentration of 5% NaCl (wt/vol) was found to be the maximum noninhibitory concentration (MNIC) (44). Siderakou et al. (44) reported that using TSAYE+5%NaCl (wt/vol) resulted in the peak of injury expressed as an increase in the time to form visible colonies, indicating that the concentration of NaCl in the selective plates poses an additional growth burden. Aliquots of 100 μl of treated samples were serially diluted in sterile Ringer’s solution and spread plated in duplicates on TSAYE (2 days, 37°C) and on TSAYE+5%NaCl (wt/vol) (5 days, 37°C). Sublethal injury after each stress treatment was determined as the difference between the numbers of colonies on TSAYE and on TSAYE+5%NaCl (wt/vol) (44), as follows:

(1) log CFU injured cells=log CFU on TSAYE − log CFU on TSAYE + 5%NaCl

The limit of detection (LOD) was 1.0 log CFU/ml for all treatments. Alterations in colony morphotype were identified based on visual inspection (macroscopic colony observation), and images were taken using a Scan 1200 HD automatic colony counter (Interscience).

### Assessment of metabolic activity with fluorescence microscopy.

In order to assess metabolic activity and potential loss of culturability, fluorescence microscopy experiments were supported by plate count experiments, using the Scott A strain. To analyze sublethal injury results, the survival of L. monocytogenes cells was evaluated comparatively (plate counting versus fluorescence microscopy) after exposure at 20°C in Ringer’s solution containing three different concentrations (20, 30, and 40 ppm) of the disinfectant PAA. Each sampling point was related to several (4 or 5) images of different fields of view (FOV), indicating metabolic activity or death.

### Staining.

In parallel with sampling at each time point, 100 μl of the stressed culture was isolated, centrifuged (13,800 × *g*, 2 min), and resuspended in 100 μl of Ringer’s solution to stop the stress exposure. Cells were stained in 10 μM 5(6)-CFDA (Sigma) solution in dimethyl sulfoxide and in 29.92 μM propidium iodide (PI) (LIVE/DEAD BacLight bacterial viability kit) solution in dimethyl sulfoxide. CFDA dyes esterase-active bacteria with intact membranes, indicating metabolic activity and producing green fluorescence. PI penetrates only cells with damaged membranes, considered dead, and produces red fluorescence. Stained cells were incubated at room temperature in the dark for 20 min. Cells were centrifuged (13,800 × *g*, 2 min) and resuspended in an equal volume of Ringer’s solution, to discard residual fluorophores. Untreated cells from the same initial colony as the treated cells were centrifuged (2,434 × *g* for 10 min at 4°C) and resuspended in 3 ml of sterile Ringer’s solution. These cultures were used to prepare positive and negative controls. Positive controls consisted of 100 μl of untreated mid-stationary-growth-phase cells in sterile Ringer’s solution stained with CFDA. As negative controls, 100-μl amounts of the above-mentioned cultures were centrifuged (13,800 × *g*, 2 min) and resuspended in 70% ethanol for 30 min at room temperature. The ethanol-killed cells were recentrifuged (13,800 × *g*, 2 min) and resuspended in 100 μl sterile Ringer’s solution, followed by staining with PI.

### Fluorescence microscopy.

Fluorescent microscopy analysis was performed using an inverted fluorescence microscope (Leica DMi8) with a DFC 7000T camera (Leica) and LAS X software (Leica). Cells were observed with an oil immersion 100× phase-contrast objective with a numeric aperture value of 1.25.

### Image analysis.

In order to analyze the microscopy images, we used a tool called BaSCA (Bacterial Single-Cell Analytics), which was developed and validated in-house (60). BaSCA is a complete pipeline that combines image processing and machine learning to achieve precise bacterial colony and single-cell segmentation, tracking, and phenotypic characterization in a fully automated mode, in terms of segmentation and morphology/expression analysis of individual cells in static and time-lapse cell movies. Briefly, the BaSCA analysis pipeline consists of image preprocessing, bacterial-colony segmentation, single-cell segmentation, cell tracking and lineage tree construction, single-cell attribute estimation, and visualization. Initially, the whole image is processed and the individual colonies are extracted. Then, each colony is analyzed and segmented into a partition of “objects” containing one or more cells until accurate single-cell boundaries are detected and cell features are estimated. Via this “divide-and-conquer” strategy, the independent analysis of different microcolonies on the images is enabled. BaSCA has been found to provide efficient analysis of microbial images (fluorescent or not) regardless of their cell density (60). Furthermore, apart from its robustness across different imaging modalities and its high level of automation, it also supports high-throughput analysis and estimation of a plethora of single-cell properties, including enumeration of cells with different fluorescent stains. The reader is encouraged to refer to Balomenos et al. for detailed information and for the validation and evaluation procedures and scores achieved by BaSCA (60).

### Statistical analysis.

The mean L. monocytogenes population (log CFU/ml) at different time points was determined with one-way analysis of variance (ANOVA) and Tukey’s honestly significant difference (HSD), using Statgraphics (version 17.2.00). The one-way analysis of variance was performed to compare the mean L. monocytogenes populations (log CFU/ml) at different stress intensities (pH values or ppm) at the same time of exposure and temperature (4°C or 20°C). Differences were considered to be significant at the 95% significance level. For pairwise comparisons between control samples (untreated cells) and the population at time zero of exposure (within 10 s), Student’s *t* test was used (Statgraphics version 17.2.00).
