# Supplementary material for: Listeria monocytogenes Sublethal Injury and Viable-but-Nonculturable State Induced by Acidic Conditions and Disinfectants
Source: Microbiol Spectr. 2021 Dec 15;9(3):e01377-21. doi: 10.1128/Spectrum.01377-21 (PMC8672913; doi:10.1128/Spectrum.01377-21)
Supplement: SUPPLEMENTAL FILE 1 — Supplemental material. Download SPECTRUM01377-21_Supp_1_seq11.pdf, PDF file, 0.7 MB [file spectrum01377-21_supp_1_seq11.pdf]

Supplementary material

Listeria monocytogenes sub-lethal injury and VBNC state induced by acidic conditions and disinfectants

Marianna Arvaniti[1], Panagiotis Tsakanikas[2], Vasiliki Papadopoulou[1], Artemis Giannakopoulou[1] and Panagiotis Skandamis[1*]

(1) Laboratory of Food Quality Control and Hygiene, Department of Food Science and Human Nutrition, Agricultural University of Athens, Athens, Greece,

(2) Laboratory of Microbiology and Biotechnology of Foods, Department of Food Science and Human Nutrition, Agricultural University of Athens, Athens, Greece

***Correspondence:** pskan@aua.gr, +30 2105294684

Keywords: *Listeria monocytogenes*; sub-lethal injury; VBNC state; acid stress; disinfectants; peracetic acid

Supplementary Figure

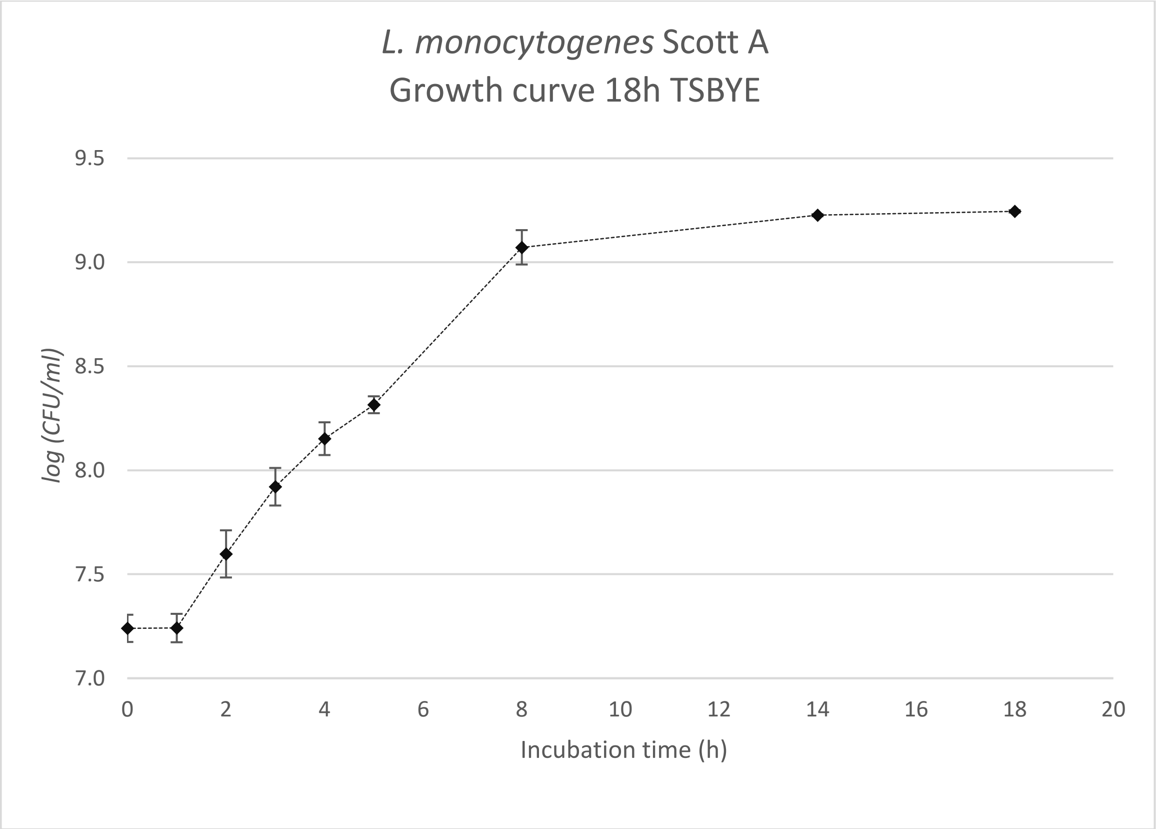

Figure S1. Population (log CFU/ml) of *L. monocytogenes*, strain Scott A, after incubation for 18h in TSBYE. Single colonies (three biological replicates tested in duplicate) from TSAYE stock culture were transferred by a loop in 10 ml TSBYE and incubated for 24h at 30°C. Following, 0.1ml of the 24h cultures were transferred in 10ml TSBYE and incubated for 18h at 30°C. Growth curve indicates that after 18h the population remains constant − in stationary phase.

Supplementary Tables

Table S1. Comparison of *L. monocytogenes* mean population (log CFU/ml) at different pH values (3.0, 2.7, 2.5) at the same time of exposure and temperature (20°C or 4°C) after exposure to AA.

	Exposure Time (h)	Total culturable population; enumerated on TSAYE			Non-injured population; enumerated on TSAYE+5%NaCl		
AA		pH 3.0	pH 2.7	pH 2.5	pH 3.0	pH 2.7	pH 2.5
20°C	0	6.90^{A}	6.99^{A}	6.43^{A}	6.85^{a}	6.99^{a}	6.30^{a}
	1	6.88^{C}	6.45^{B}	<LOD^{A}	6.82^{b}	5.98^{b}	<LOD^{a}
	2	6.56^{C}	3.28^{B}	<LOD^{A}	6.54^{c}	2.41^{b}	<LOD^{a}
	3	5.86^{B}	<LOD^{A}	<LOD^{A}	5.42^{b}	<LOD^{a}	<LOD^{a}
	4	4.83^{B}	<LOD^{A}	<LOD^{A}	3.81^{b}	<LOD^{a}	<LOD^{a}
	5	3.08^{B}	<LOD^{A}	<LOD^{A}	2.26^{b}	<LOD^{a}	<LOD^{a}
4°C	0	6.76^{A}	6.90^{A}	6.81^{A}	6.75^{c}	6.91^{b}	6.41^{a}
	1	6.68^{B}	6.19^{AB}	5.94^{A}	6.73^{b}	5.59^{b}	1.66^{a}
	2	6.61^{C}	3.72^{A}	5.09^{B}	6.65^{c}	2.66^{b}	<LOD^{a}
	3	6.53^{B}	3.04^{A}	3.61^{A}	6.48^{b}	<LOD^{a}	<LOD^{a}
	4	6.46^{B}	2.66^{A}	2.32^{A}	6.23^{b}	<LOD^{a}	<LOD^{a}
	5	6.27^{B}	1.46^{A}	<LOD^{A}	5.76^{b}	<LOD^{a}	<LOD^{a}

Statistically significant differences ($p < 0.05$, CI 95%) are indicated with different uppercase (TSAYE) and lowercase (TSAYE-5%NaCl) letters, respectively.

Table S2. Comparison of *L. monocytogenes* mean population (log CFU/ml) at different pH values (3.0, 2.7, 2.5) at the same time of exposure and temperature (20°C or 4°C) after exposure to HCl.

	Exposure Time (h)	Total culturable population; enumerated on TSAYE			Non-injured population; enumerated on TSAYE+5%NaCl		
HCl		pH 3.0	pH 2.7	pH 2.5	pH 3.0	pH 2.7	pH 2.5
20°C	0	7.13^{A}	6.93^{A}	7.06^{A}	7.10^{a}	6.92^{a}	6.96^{a}
	1	7.17^{C}	6.78^{B}	6.24^{A}	7.07^{b}	6.73^{b}	5.85^{a}
	2	7.12^{C}	5.65^{B}	2.72^{A}	7.07^{c}	5.39^{b}	2.64^{a}
	3	7.09^{C}	4.27^{B}	<LOD^{A}	7.02^{c}	4.36^{b}	<LOD^{a}
	4	7.12^{C}	3.54^{B}	<LOD^{A}	6.99^{c}	3.52^{b}	<LOD^{a}
	5	7.08^{C}	3.09^{B}	<LOD^{A}	6.97^{c}	2.94^{b}	<LOD^{a}
4°C	0	6.99^{A}	7.15^{AB}	7.07^{B}	7.01^{a}	7.16^{ab}	7.05^{b}
	1	6.93^{A}	7.03^{A}	6.51^{A}	6.94^{a}	7.05^{a}	6.22^{a}
	2	6.91^{B}	7.06^{B}	5.19^{A}	6.91^{b}	7.05^{b}	4.61^{a}
	3	6.81^{B}	6.98^{B}	3.69^{A}	6.82^{b}	6.98^{b}	3.22^{a}
	4	6.78^{C}	6.95^{B}	2.79^{A}	6.83^{b}	6.90^{b}	2.69^{a}
	5	6.82^{B}	6.90^{B}	2.09^{A}	6.72^{b}	6.84^{b}	2.01^{a}

Statistically significant differences ($p < 0.05$, CI 95%) are indicated with different uppercase (TSAYE) and lowercase (TSAYE-5%NaCl) letters, respectively.

Table S3. Comparison of *L. monocytogenes* mean population (log CFU/ml) at different ppm (0.5, 5, 10, 20, 30, 40) at the same time of exposure and temperature (20°C or 4°C) after exposure to PAA.

PAA	Exposure Time (sec)	Total culturable population; enumerated on TSAYE log (CFU/ml)						Non-injured population; enumerated on TSAYE+5%NaCl log (CFU/ml)					
		0.5ppm	5ppm	10ppm	20ppm	30ppm	40ppm	0.5ppm	5ppm	10ppm	20ppm	30ppm	40ppm
20°C	0	7.09^{A}	6.93^{A}	6.76^{A}	6.57^{A}	6.77^{A}	6.25^{A}	6.98^{c}	6.91^{c}	6.75^{bc}	5.79^{ab}	5.37^{a}	5.19^{a}
	30	7.10^{B}	6.87^{B}	6.80^{B}	5.13^{A}	5.03^{A}	4.89^{A}	7.03^{c}	6.84^{c}	6.70^{c}	4.11^{b}	3.10^{a}	3.50^{ab}
	60	7.11^{B}	6.83^{B}	6.72^{B}	3.94^{A}	4.56^{A}	3.20^{A}	7.10^{c}	6.80^{c}	6.49^{c}	3.13^{b}	1.19^{a}	1.62^{ab}
	300	7.16^{D}	4.78^{C}	3.86^{C}	2.37^{B}	4.04^{C}	<LOD^{A}	7.08^{d}	3.19^{c}	2.58^{bc}	1.74^{ab}	<LOD^{a}	<LOD^{a}
	900	7.07^{B}	<LOD^{A}	<LOD^{A}	1.44^{A}	<LOD^{A}	<LOD^{A}	6.95^{b}	<LOD^{a}	<LOD^{a}	<LOD^{a}	<LOD^{a}	<LOD^{a}
	1800	7.02^{B}	<LOD^{A}	<LOD^{A}	<LOD^{A}	<LOD^{A}	<LOD^{A}	6.93^{b}	<LOD^{a}	<LOD^{a}	<LOD^{a}	<LOD^{a}	<LOD^{a}
4°C	0	7.08^{AB}	7.05^{AB}	7.15^{B}	6.59^{A}	6.96^{AB}	6.88^{AB}	7.03^{a}	7.05^{a}	7.14^{a}	6.54^{a}	6.80^{a}	6.60^{a}
	30	7.10^{A}	7.02^{A}	6.96^{A}	6.81^{A}	6.87^{A}	6.12^{A}	7.11^{a}	6.95^{a}	6.96^{a}	6.32^{a}	6.47^{a}	5.77^{a}
	60	7.10^{A}	6.99^{A}	7.05^{A}	6.37^{AB}	6.57^{AB}	4.39^{A}	7.09^{b}	6.92^{b}	6.99^{b}	5.21^{b}	5.26^{b}	1.45^{a}
	300	7.12^{A}	6.77^{A}	6.29^{A}	2.83^{B}	<LOD^{A}	<LOD^{A}	7.07^{c}	6.74^{c}	4.69^{b}	<LOD^{a}	<LOD^{a}	<LOD^{a}
	900	7.07^{C}	6.72^{C}	3.59^{B}	<LOD^{A}	<LOD^{A}	<LOD^{A}	7.04^{c}	6.61^{b}	<LOD^{a}	<LOD^{a}	<LOD^{a}	<LOD^{a}
	1800	7.07^{B}	6.73^{B}	1.24^{A}	<LOD^{A}	<LOD^{A}	<LOD^{A}	7.01^{c}	6.47^{b}	<LOD^{a}	<LOD^{a}	<LOD^{a}	<LOD^{a}

Statistically significant differences ($p < 0.05$, CI 95%) are indicated with different uppercase (TSAYE) and lowercase (TSAYE-5%NaCl) letters, respectively.

Table S4. Comparison of *L. monocytogenes* mean population (log CFU/ml) at different ppm (20, 30, 40) at the same time of exposure and temperature (20°C or 4°C) after exposure to PAA.

	Exposure time	Total culturable population; enumerated on TSAYE			Non-injured population; enumerated on TSAYE+5%NaCl		
20°C	(h)	20ppm	30ppm	40ppm	20ppm	30ppm	40ppm
PAA	0	9.17^{C}	5.29^{B}	3.05^{A}	8.88^{c}	4.14^{b}	2.61^{a}
	1	9.14^{C}	4.57^{B}	2.85^{A}	8.80^{c}	3.77^{b}	2.14^{a}
	2	9.10^{C}	3.72^{B}	2.72^{A}	8.86^{c}	3.39^{b}	1.59^{a}
	3	9.13^{C}	1.59^{B}	$<LOD^{A}$	8.90^{c}	1.39^{b}	$<LOD^{a}$

Statistically significant differences ($p < 0.05$, CI 95%) are indicated with different uppercase (TSAYE) and lowercase (TSAYE-5%NaCl) letters, respectively.
